# Uptake of MicroRNAs from Exosome-Like Nanovesicles of Edible Plant Juice by Rat Enterocytes

**DOI:** 10.3390/ijms22073749

**Published:** 2021-04-03

**Authors:** Yuko Ito, Kohei Taniguchi, Yuki Kuranaga, Nabil Eid, Yosuke Inomata, Sang-Woong Lee, Kazuhisa Uchiyama

**Affiliations:** 1 Department of General and Gastrological Surgery, Osaka Medical College, 2-7, Takatsuki, Osaka 569-8686, Japan; kohei.taniguchi@ompu.ac.jp (K.T.); yosuke.inomata@ompu.ac.jp (Y.I.); sang-woong.lee@ompu.ac.jp (S.-W.L.); uchi@ompu.ac.jp (K.U.); 2 Translational Research Program, Osaka Medical College, 2-7, Daigaku-cho, Takatsuki, Osaka 569-8686, Japan; 3 United Graduate School of Drug Discovery and Medical Information Sciences, Gifu University, 1-1, Yanagito, Gifu 501-1193, Japan; yk.kuranaga@gmail.com; 4 Department of Anatomy, College of Medicine and Health Sciences, United Arab Emirates University, P.O. Box 17666 Al Ain, United Arab Emirates; nabileidm@uaeu.ac.ae

**Keywords:** edible plant, enterocyte, exosome, extracellular vesicle, microRNA

## Abstract

MicroRNAs (miRNAs) are small RNAs present in extracellular vesicles (EVs) that, when transferred to a target cell, affect its biological functions. Plant miRNAs regulate the expression of certain mammalian genes. Here, we characterized EVs in fruit and vegetable juice, and their miRNA cargo, and investigated whether such miRNA-containing EVs could be taken up by mammalian enterocytes in vitro. Using filtration and ultra-centrifugation methods, EVs were purified from commercially available and manually squeezed plant juice. EV morphological features and subcellular localization were analyzed using the NanoSight tracking system and electron microscopy. Plant EV miRNA levels were evaluated using quantitative reverse transcription PCR. For the in vitro EV uptake experiments, rat intestinal epithelial cells (IEC6) were used. Plant EVs shared morphological features with mammalian EVs and contained miR156a-5p, miR166a-3p, and miR168a-5p. EVs were present in the cell sap-filled central vacuoles and were taken up by IEC6 cells. Edible plant cells produce EVs that contain various miRNAs and release them into the central vacuole. The exogenous plant EVs are taken up by mammalian enterocytes in vitro. These findings suggest the possibility that exogenous plant miRNAs carried by EVs can be absorbed via the gastrointestinal tract.

## 1. Introduction

MicroRNAs (miRNAs) are small non-coding RNA molecules that are, on average, 25 nucleotides long. They post-transcriptionally control the expression of the target gene by binding to the 3′-untranslated region of mRNA. MiRNAs from plant and animal food are absorbed by mammals [1]. As recently discovered, such food-derived miRNAs are involved in cross-kingdom gene regulation [2]. For example, plant miR168a targets the expression of the mammalian low-density lipoprotein receptor adapter protein 1 [3].

Mammalian cells secrete extracellular vesicles (EVs), which are intercellular communication tools and contain functional nucleic acids (mRNA, miRNA, or other RNA species). EVs are categorized into the main exosome, which has an endosomal origin and is approximately 50–100 nm in diameter, and microvesicles, which bud from the cytoplasm and are approximately 100–1000 nm in diameter [4,5]. By contrast, compared with mammalian EVs, little is known about plant miRNAs and plant EVs [6]. In addition, the function, origin, formation, and secretion of plant EVs and EV markers, which are crucial for their detection, are not well researched [7,8]. Of note, the presence of plant miRNAs in the plasma of plant-eating mammals has been reported [9,10]. Exogenous plant miRNAs and/or EVs are primarily taken up orally and are found in the mammalian serum, urine, and feces; however, their transport to the gastrointestinal (GI) tract and incorporation into cells therein are unclear [2,3,11,12].

However, several lines of evidence suggest that plants also release EVs having various functions including symbiosis and defense against pathogens [13]. EVs from different eukaryotic and prokaryotic organisms are involved in many processes, such as host–pathogen interactions, resistance transmission, and plant diseases, which may help in the prevention and treatment of pests and pathogens [14]. Interestingly, a growing body of evidence indicates that autophagy-related proteins are involved in exosome biogenesis, which indicates that there are pathways for the interaction between autophagy and EVs that may be important in the context of cancer and neurodegenerative diseases [15].

We hypothesized that EVs present in plant juice act as a cargo defense to protect plant miRNAs from gastric juices and RNases. MiRNAs packed into EVs would be transported to the GI tract, taken up by intestinal enterocytes by endocytosis, and then transported into the circulation and organs. Hence, we analyzed the morphological features and subcellular localization of EVs in plant juice using electron microscopy and nano-tracking analysis (NTA). We then showed that plant EVs contain ubiquitous plant miRNAs and are taken up by rat intestinal enterocytes. These findings suggest that plant miRNAs present in edible juice can be absorbed in the GI tract, opening the possibility of using such EVs as a drug delivery system and for gene therapy with plant miRNAs.

## 2. Results

### 2.1. Vegetable and Fruit Juices Contain EVs

**Figure 1:**
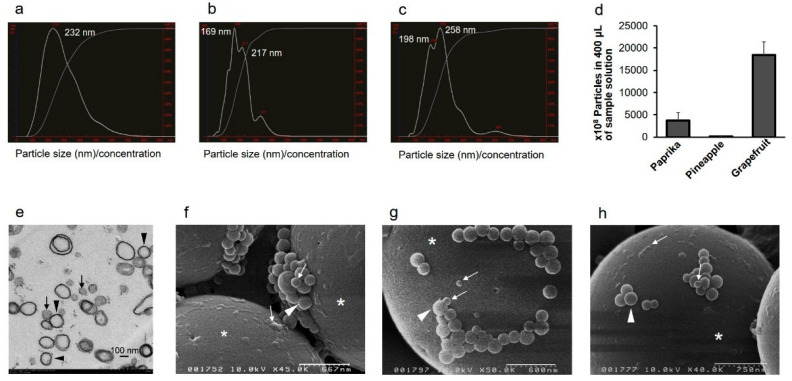
Size characterization of extracellular vesicles (EVs) from fruit and vegetable juice. (**a**–**d**) NanoSight analysis of EVs. Representative particle size distributions for EVs are as follows: paprika juice (freshly hand-squeezed juice, SJ) (**a**), pineapple juice (commercially available 100% natural fruit juice, J) (**b**), and grapefruit J (**c**). (**d**) Number of EVs in juice samples. The data are shown as average ± standard deviation (*n* = 3). Paprika SJ: 3738 (1815); pineapple J: 105 (15); grapefruit J: 18,467 (2902). (**e**) Transmission electron microscopy analysis of an EV pellet from grapefruit J. Exosome-like nanovesicles are visible, approximately 50–100 nm (black arrows) and 200 nm (black arrowheads) in diameter. Bar = 100 nm. (**f**–**h**) Scanning electron microscopy analysis of EVs. Exosome-like nanovesicles from paprika SJ (**f**), pineapple J (**g**), and grapefruit J (**h**) are shown. The vesicles are approximately 50–100 nm (white arrows) and 200 nm (white arrowheads) in diameter. White asterisks: 3 μm polyethylene beads. Scale bars: f, 667 nm; g, 600 nm; h, 750 nm.

To characterize EVs in plant juice, NTA was first used to measure the size of plant-derived EVs in paprika juice (freshly hand-squeezed juice, SJ), pineapple juice (commercially available 100% natural fruit juice, J), and grapefruit J. The EVs were approximately 50–200 nm in diameter. The mode size of vesicles was 232 ± 114 nm in paprika SJ, 169 ± 70 nm in pineapple J, and 258 ± 104 nm in grapefruit J (Figure 1a–c). The EV count was the highest in grapefruit J and the lowest in pineapple J (Figure 1d). Scanning electron microscopy (SEM) analysis revealed the presence of exosome-like particles, approximately 50–200 nm in diameter, in these juices (Figure 1f–h). Similarly, transmission electron microscopy (TEM) analysis of EV pellets from grapefruit J revealed the presence of exosome-like vesicles (Figure 1e). These observations confirmed the presence of EVs in fruit and vegetable juice.

### 2.2. Epidermal Cells Contain EVs

**Figure 2:**
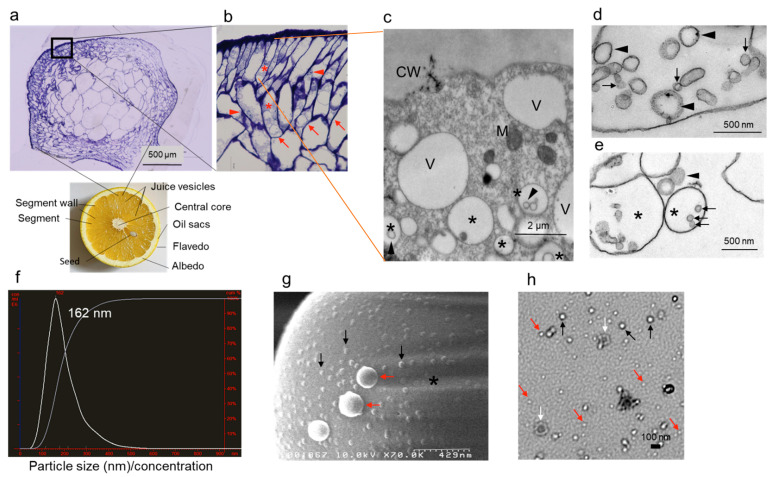
Subcellular localization and size characterization of EVs in epidermal cells of grapefruit juice vesicle. (**a**,**b**) Modified structure of cross sections of grapefruits and semi-thin sections of grapefruit juice vesicles. Juice vesicles are shown in segment (**a**). Numerous vacuoles (red arrows) are visible in the epidermal cells (red asterisks). The nuclei of epidermal cells are denoted by red arrowheads. Image in (**b**) is an enlarged view of image in (**a**). Black box: the enlarged area. Bar = 500 μm. (**c**–**e**) Transmission electron microscopy (TEM) analysis of epidermal cells. (**c**) Multivesicular bodies (MVBs) (black asterisks) contain exosome-like nanovesicles, which are approximately 50–100 nm in diameter (black arrowheads). CW, cell wall; M, mitochondrion; V, vacuoles. Bar = 2 μm. (**d**) Exosome-like nanovesicles, approximately 50–100 nm (black arrow) and 200 nm (black arrowheads) in diameter, in the central vacuole. Bar = 500 nm. (**e**) MVBs (black asterisks) and exosome-like nanovesicles in a vacuole, approximately 50–100 nm (black arrows) and 200 nm (black arrowheads) in diameter. Bar = 500 nm. (**f**) NanoSight analysis of grapefruit juice (freshly hand-squeezed juice, SJ). Representative particle size distribution for grapefruit SJ nanovesicle measurements is shown. (**g**) Scanning electron microscopy analysis of exosome-like nanovesicles in grapefruit SJ, approximately 50–100 nm (black arrows) and 200 nm (red arrows) in diameter. Bar = 429 nm. Black asterisks: 3 μm polyethylene beads. (**h**) TEM analysis of exosome-like nanovesicles in grapefruit SJ. Black arrows: vesicles of approximately 50–100 nm in diameter; red arrows: smaller exosome-like nanovesicles (less than 50 nm in diameter); white arrows: vesicles that are approximately 200 nm in diameter. Bar = 100 nm.

To investigate the source of EVs in juice, grapefruit juice vesicles were sectioned and analyzed. Analysis of semi-thin sections of juice vesicles revealed the presence of many vacuoles in epidermal cells (Figure 2a,b). The cytoplasm of epidermal cells contained two types of vacuoles: empty vacuoles and vacuoles filled with vesicles (multivesicular bodies, MVBs) (Figure 2c). Central vacuoles contained many EV-containing MVBs (Figure 2d,e). Based on the NTA, the mode size of exosome-like particles in grapefruit SJ was 162 ± 76 nm (Figure 2f). SEM and TEM analysis confirmed that exosome-like vesicles were abundant in grapefruit SJ (Figure 2g,h). Smaller vesicles (less than 50 nm in diameter) were also observed in grapefruit SJ (Figure 2h). These analyses indicate that epidermal cells of grapefruit juice vesicles contain EVs.

### 2.3. Plant Juice EVs Contain Plant MiRNAs

Given the cross-kingdom miRNA trafficking between plants and animals [3], we focused on miR168a, miR166a, and miR156a, which are commonly found in plant cells and in edible plant J and SJ. Total RNA was isolated from EVs of pineapple J, grapefruit J, paprika SJ, and grapefruit SJ and analyzed using quantitative RT-PCR. The analysis confirmed that the EVs contained miR156a-5p, miR166a-3p, and miR168a-5p (Table 1). Grapefruit SJ and paprika SJ contained more miRNAs than grapefruit J and pineapple J, with moderate miR168a-5p and miR166a-3p levels in grapefruit SJ and paprika SJ. These differences might be associated with the difference in SJ and J processing, namely made manually or in the factory.

### 2.4. EVs Containing Plant MiRNAs Are Taken Up by Intestinal Epithelial Cells (IEC6)

**Figure 3:**
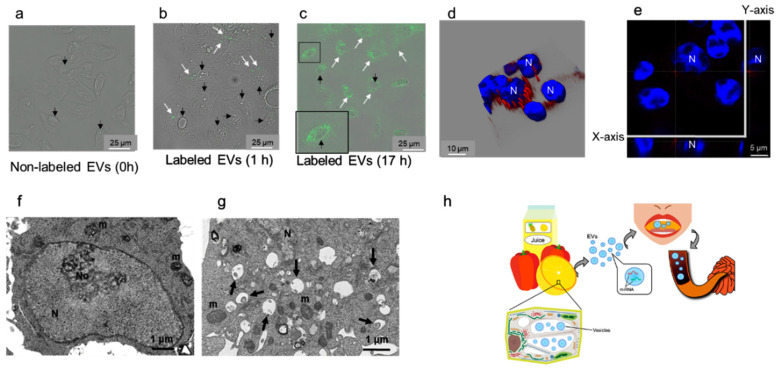
Plant extracellular vesicle (EV) uptake by intestinal epithelial cells (IEC6) and summary of the main findings of the current study. (**a**–**e**) Confocal laser scanning microscopy analysis of IEC6 cell interaction with plant EVs. (**a**) IEC6 cells exposed to unlabeled EVs for control (0 h). IEC6 cells after 1 h (**b**) and 17 h (**c**) of exposure to EVs labeled with PKH67 (green). White arrows: EVs labeled with PKH67 taken up by IEC6 cells. Black arrows: nuclei of IEC cells are shown with differential interference contrast (DIC). The inset in c shows an enlarged boxed area. Bar = 25 μm. IEC6 cells after 17 h (**d**,**e**) of exposure to EVs labeled with PKH26 (red). N, nucleus stained with 4′,6-diamidino-2-phenylindole (blue). A three-dimensional composite image of *Z*-sections of IEC6 cells (**d**) and one *Z*-section from (**d**) with the X and Y sections indicated (**e**) are shown. Perinuclear localization of labeled EVs is apparent. (**f**,**g**) Transmission electron microscopy analysis of IEC6 cell interaction with plant EVs. (**f**) IEC6 cells were exposed to phosphate-buffered saline (PBS) instead of EVs. (**g**) IEC6 cells after 17 h of exposure to EVs. Endosomes and phagosomes (black arrows) are localized in the perinuclear region. N, nucleus; No, nucleolus; m, mitochondria. (**h**) Overview of the implications of the findings of the current study. Extracellular vesicles are present in the multivesicular bodies (MVBs) and central vesicles in plant epidermal cells. Juices squeezed from edible plants and 100% natural edible plant juice available on the market contain EVs that correspond to exosome-like nanovesicles. These nanovesicles contain microRNAs (miRNAs). When humans drink juice, miRNAs (packed in EVs to protect them from degradation) pass through the gastrointestinal tract and are absorbed by small intestine enterocytes.

Rat IEC6 cells were exposed to PKH fluorescent-labeled EVs from grapefruit SJ. In the experiment, a control experiment that showed no phagosome containing PKH67-labeled EVs (Figure 3a), EVs were incorporated into the cells within 15 min, the number of EVs labeled with PKH67 (visualized as green fluorescent dots using fluorescence microscopy) in rat cells increased with time, and EVs translocated to the perinuclear region of IEC6 cells after 17 h (Figure 3a–c). Incorporation of EVs labeled with PKH26 was also analyzed using a three-dimensional analysis, which indicated that EVs were localized in the cytoplasm, especially in the perinuclear area of IEC6 cells (Figure 3d,e). Based on the TEM analysis, PKH-labeled grapefruit SJ EVs were endocytosed and/or phagocytosed by IEC6 cells (Figure 3f,g). Further, the levels of miR168a-5p in IEC6 cells exposed to EVs from grapefruit SJ were significantly higher than those in IEC6 cells exposed to phosphate-buffered saline (Table 2). Collectively, these findings indicate that IEC6 cells take up miRNA-containing EVs present in grapefruit SJ.

## 3. Discussion

In the current study, we aimed to characterize EVs in plant juice and to understand whether they can be taken up by mammalian cells. We demonstrated that commercially available fruit juice and freshly squeezed fruit and vegetable juice contain EVs. These EVs exhibit the same morphological features as those of mammalian exosomes and contain ubiquitous plant miRNAs, namely miR156a-5p, miR166a-3p, and miR168a-5p. Further, we detected exosome-like EVs in MVBs and central vacuoles containing large amounts of cell sap, which were drunk together with juice by humans. We also showed that mammalian small intestine epithelial cells uptake plant EVs containing miRNAs. These findings indicate that exogenous plant miRNAs can be absorbed via the GI tract upon ingestion.

Nanoparticles or exosome-like nanoparticles derived from grapefruit have been successfully used as delivery tools for therapeutic agents [16,17,18]. According to the previous studies, the average nanoparticle size is 50–1000 nm, and the particles are made up of grapefruit-derived lipids. Here, we showed that the structure of EVs from plant juice (Figure 1 and Figure 2) is similar to that of mammalian exosomes [19,20,21]. Specifically, the size of EVs from plant juice was 50–200 nm, which is the same as that of mammalian cells.

The presence of MVBs and a paramural body associated with the cell wall was previously demonstrated in barley leaf cells attacked by the biotrophic powdery mildew fungus using TEM [7]. MVBs move into the central vacuole to endocytose vesicles for degradation, and the paramural body delivers vesicles to the extracellular space via membrane fusion with the plasma membrane as a defense response against pathogen attack [7,22]. Similarly, we observed MVBs, structures with intraluminal vesicles, in the cytoplasm near the plasma membrane and central vacuoles in grapefruit epidermal cells (Figure 2c,d).

Previously constructed miRNA libraries contain the sequences of 26 conserved miRNAs frequently found in exosome-like nanoparticles derived from 11 edible plants [23]. The levels of these 26 miRNAs are significantly higher than those of the other 39, moderately present, miRNAs [23]. Here, we detected miR156a-5p, 166a-3p, and 168a-5p, which are frequently observed miRNAs [23], in exosome-like EVs derived from grapefruit, paprika, and pineapple juice (Table 1). Their levels in grapefruit SJ and paprika SJ were higher than those in grapefruit J and pineapple J (Table 1). Although the concentration of grapefruits SJ (339.6 × 108 mL) was lower than that of grapefruits J (Figure 1d), the expression of miRNAs for grapefruits SJ was higher than that of grapefruits J (Table 1). We hypothesize that EVs act to protect the miRNA cargo, and, hence, miRNAs are well preserved in EVs in hand-squeezed juice as opposed to factory-produced, commercially available juice.

Exogenous plant miRNAs were detected in human and animal serum and plasma, and food-derived plant miR168a can pass through the mouse GI tract and enter the circulation and liver [3]. In a mouse oral gavage experiment, plant exosome-like nanoparticles were found in stem cells and macrophages of the submucosa of the small intestine 6 h after administration [11]. In addition, oral intake of ginger-derived nanoparticles mediates the activation of nuclear factor erythroid 2-related factor 2 in hepatocytes of ethanol-treated mice [24]. In another in vitro experiment, enterocyte uptake of plant miRNAs and repackaging into microvesicles (recently redefined as exosomes) was documented [3]. EVs act as a small-cargo defense, protecting miRNAs from degradation by RNases or digestive juices. By contrast, exogenous plant miRNAs, which are EV-free, were detected in animal blood, the GI tract, various organs (the kidney, liver, and spleen), and feces after oral gavage in a mouse model [3,12]. Further, in the nematode *Caenorhabditis elegans*, systemic RNA interference defective transmembrane protein 1 (SIDT-1) and SIDT-2 might mediate the luminal membrane passage of double-stranded RNA through the GI tract [25,26,27,28]. Furthermore, extracellular plant miRNAs and single-stranded RNA associated with Argonaute protein family 2 are taken up by enterocytes expressing SIDT family proteins [3,25,29]. In the current study, TEM analysis indicated that mammalian enterocytes endocytose and/or phagocytose plant EVs. This results in the perinuclear EV localization in the cytoplasm (Figure 3f,g) and presence of plant miRNAs in the cell (Table 2). Further studies, especially using in vivo systems, are needed to demonstrate the fate of EVs incorporated into enterocytes.

In the current study, we were able to separate plant exosome-like nanovesicles from fruits and vegetables because they are approximately 50–200 nm in diameter, a size that is the same as that of mammalian exosomes previously studied by us [19,20,21]. MVBs, in addition to their association with the plasma membrane, were localized in the central vacuole (Figure 2). We similarly separated nanovesicles from commercially available fruit and vegetable juice (Figure 1). In addition to size-based identification, molecular markers for plant EVs are being researched [8,22]. Several such markers have already been identified for mammalian EVs [19,20,21], but further biochemical studies to detect plant EV markers are needed.

It is generally accepted that “juice” consists of cell sap of an edible plant. Indeed, here we purified plant EVs from the cell sap. These EVs contained ubiquitous plant miRNAs (Table 1). Although we used primers specific to miR156a-5p, miR166a-3p, and miR168a-5p, which were adjusted for *Citrus sinensis*, we were able to detect these miRNA levels in other species. We thus concluded that these miRNAs are ubiquitous plant miRNAs. In addition, using a sodium-periodate-based method for the detection of plant miRNAs might be better suited to plant miRNAs than using the general method because the plant miRNA terminal nucleotide of these miRNAs is 2′-*O*-methylated [30].

Several limitations of the current study should be acknowledged. First, we did not use any of the plant EV markers, such as Rab11a and TET8,9, that have been suggested in previous studies [8,22], nor detected the expression of proteins. We mainly detected plant EVs based on morphology. Further experiments are needed to more easily observe plant EVs. Second, the EV uptake analysis relied on an in vitro approach. We hypothesize that mammalian enterocytes endocytose and/or phagocytose plant EVs because of their ability to absorb nutrients (Figure 3h). However, although we observed the uptake of EVs by enterocytes, we did not investigate the possibility of miRNA secretion into the circulation and/or lymph in vivo. An animal gavage with plant EVs would enable us to evaluate any cross-kingdom gene regulation by plant miRNAs in more detail.

## 4. Materials and Methods

### 4.1. Isolation of Plant EVs

EVs were purified from pineapple J, grapefruit J, grapefruit SJ, and paprika SJ by centrifugation at 2000× *g* for 5 min. For SJ, segments were squeezed through filter papers. The supernatant was filtered through a 0.45 μm pore filter to remove cellular debris and then through a 0.2 μm pore filter to isolate exosome-sized EVs. The flow-through fraction was ultra-centrifuged at 100,000× *g* for 4 h. The supernatant was then carefully removed, and the pellet was used in the ensuing experiments.

### 4.2. NTA

NTA measurements were performed using NanoSight LM10 (NanoSight, Wiltshire, UK). The EV pellets were suspended in 1 mL of 0.1 M PBS and analyzed using an NTA Version 2.3 Build 0034 instrument (NanoSight). The following photographic settings were used: frames processed, 1498 of 1498 or 1499 of 1499; frames per second, 24.97 or 24.98; calibration, 190 nm/pixel; and detection threshold, 6 or 7 multi.

### 4.3. SEM

Plant EV pellets suspended in PBS were fixed in 1.25% (*v*/*v*) glutaraldehyde (pH 7.4) for 15 min and then placed on 3 μm polyethylene beads (White Estapor^®^ Microspheres; Merck Chimie S.A.S., Lyon, France) coated with poly-L-lysine. Specimens were rinsed in PBS, post-fixed in 1% (*v*/*v*) osmium tetroxide (pH 7.4) for 20 min, and dehydrated in a graded ethanol series. After dehydration, samples were coated with platinum–palladium and observed using SEM (Hitachi S-5000; Hitachi, Tokyo, Japan).

### 4.4. TEM

EV pellets from grapefruit SJ were fixed in 2.5% glutaraldehyde (pH 7.4) for 40 min, post-fixed in 1% osmium tetroxide (pH 7.4) for 40 min, and then dehydrated in a graded ethanol series. After dehydration, samples were embedded in an epoxy resin mixture, polymerized, cut into 70 nm sections, and then observed using TEM (Hitachi H-7100 and H-7650 instruments; Hitachi). To observe EVs in grapefruit epidermal cells, grapefruit juice vesicles were prepared in the same manner as pellets. To observe the whole EV structure, EVs were stained using a modified negative staining method on grids coated with Excell support film (Nisshin EM, Tokyo, Japan) and lead solution.

### 4.5. Plant EV Uptake by Rat Intestine Epithelial Cells

EVs purified from grapefruit SJ were labeled with PKH67 or PKH26 using Fluorescent Cell Linker kits (Sigma-Aldrich, St. Louis, MO, USA), according to the manufacturer’s instructions. The rat intestinal epithelial cell line IEC6 was purchased from RIKEN BRC CELL BANK (Tsukuba, Japan) and cultured under 5% CO_2_ at 37 °C in an incubator. For the experiments, 5–10 μg of PKH-labeled EVs or EVs pre-incubated with PBS instead of PKHs as a control were added to cultured IEC6 enterocytes and incubated at 37 °C for 15 min, 1 h, 6 h, or 17 h. Then, the cells were analyzed using confocal laser scanning microscopy (TCS SP8; Leica, Wetzlar, Germany) and/or TEM. Plant miRNAs in IEC6 cells were also detected using quantitative RT-PCR (as described in Section 4.6).

### 4.6. Quantitative Reverse Transcription PCR (qRT-PCR)

Total RNA from plant EVs was isolated using the mirVana miRNA Isolation Kit (Invitrogen, Carlsbad, CA, USA), according to the manufacturer’s protocol. To detect miRNA levels in EVs from plant cells, quantitative RT-PCR was performed using the TaqMan^®^ MicroRNA Reverse Transcription Kit (Applied Biosystems, Foster City, CA, USA) and THUNDERBIRD Probe qPCR Mix (TOYOBO Co., Ltd., Osaka, Japan), according to the manufacturers’ protocols. Primers targeting miR156a-5p, miR166a-3p, and miR168a-5p from *C. sinensis* were purchased from TaqMan^®^ MicroRNA Assays (Applied Biosystems). The relative miRNA levels were calculated using the ΔΔCt method. To verify the presence of plant miRNAs in IEC6 cells in the uptake experiment, total RNA was extracted using the mirVana miRNA Isolation Kit (Invitrogen), and miR168a-5p levels were detected using Takara TP870 (Takara Bio Inc., Kusatsu, Japan).

### 4.7. Terminology

The miRNA terminology used herein is based on that of the Plant MiRNA Database (http://bioinformatics.cau.edu.cn/PMRD/; accessed on 9th September 2020) [31].

### 4.8. Statistical Analysis

Data analysis was performed using StatMate III software (ATMS, Co., Ltd., Chiba, Japan), with the Student’s *t*-test used to compare continuous variables between groups. *p* values of 0.05 were considered statistically significant. Data are expressed as means and standard deviations.

## 5. Conclusions

In conclusion, we have purified exosome-like nanovesicles from fruit and vegetable juice, the cell sap. The EVs contain ubiquitous plant miRNAs, and their morphological features are similar to those of mammalian EVs. Further, we showed the uptake of plant exosome-like nanovesicles containing miRNAs by mammalian small intestine enterocytes in vitro. These observations suggest that exogenous plant miRNAs can be absorbed via the GI tract upon ingestion. Furthermore, our findings suggest that exogenous plant miRNAs that have therapeutic effects on human health might be administered orally.

## Figures and Tables

**Table 1:** Threshold cycle values of different microRNAs in extracellular vesicles extracted from fruit and vegetable juices.

Gene	Grapefruit SJ	Grapefruit J	Paprika SJ	Pineapple J
*MIR156a-5p*	28.96 ± 0.04	33.69 ± 0.246	30.6 ± 0.2	39.5 ± 0.42
*MIR166a-3p*	29.11 ± 0.011	33.33 ± 0.001	23.56 ± 0.08	44.36 ± 4.92
*MIR168a-5p*	26.29 ± 0.17	31.86 ± 0.09	25.62 ± 0.07	38.68 ± 0.88

J, Commercially available 100% natural fruit juice; SJ, freshly hand-squeezed juice. The data are shown as average ± standard deviation (*n* = 6).

**Table 2:** Threshold cycle values of *MIR168* in grapefruit EVs and intestinal epithelial cells (IEC6) after administration of grapefruit EVs.

Gene	Grapefruit EVs	IEC6-PBS	IEC6-GFEVs
*MIR168a-5p*	25.18 ± 0.69	37.15 ± 0.57	35.50 ± 0.39 **

** *p* < 0.001 (Student’s *t*-test). IEC6-PBS, cells exposed to PBS as a control; IEC6-GFEVs, cells exposed to grapefruit EVs. The data are shown as average ± SD (*n* = 6).

## Data Availability

Not applicable.
